# Calendar

**DOI:** 10.1155/S1463924683000309

**Published:** 1983


Journal of Automatic Chemistry, Volume 5, Number 2 (April–June 1983), pages 112–113

### Calendar

**Editor's note**
*Organizers of conferences, seminars, etc. should send details for inclusion in the calendar as soon as the relevant information is available and not later than three months before the event.*

### May 1983

**Biotechnology for Fuels and Chemicals: Fifth Oak Ridge National Laboratory Symposium**
To be held from 10 to 13 May in Gatlinburg, Tennessee, USA.
*Further information from Charles D. Scott, Chemical Technology Division, Oak Ridge National Laboratory, PO Box X, Oak Ridge, Tennessee 37830, USA.*

**Haemonetics Research Laboratory: Tenth International Advanced Apheresis Seminar**
To be held from 16 to 17 May in Boston, Massachusetts, USA.
*Further information from Douglas A. Moe, Haemonetics Research Institute, 400 Wood Road, Braintree, Massachusetts 02184, USA.*

### June 1983

**Hands-On GC$^2$**
Course to be held from 6–7 and 8–9 June at the Chemistry Department, Brunel University, Uxbridge, UK.
*Further information from Charles D. Cook, Erba Science (UK) Ltd, Headlands Trading Estate, Swindon, Wiltshire SN2 6JQ, UK.*

**1983 International Gas Research Conference**
To be held from 13 to 16 June in London.
*Further information from Conference Services Ltd, 3 Bute Street, London SW7 3EY.*

**Tetronica 83**
To be held at Earls Court in London from 14–17 June 1983.
*Further information from Industrial & Trade Fairs Ltd, Radcliffe House, Blenheim Court, Solihull, West Midlands B91 2BG, UK.*

**Modern Methods of Food Analysis**
A basic science symposium organized by the Institute of Food Technologists. To be held from 17–18 June in New Orleans, USA.
*Further information from Dr John Whitaker, University of California, Davis, California 95616, USA.*

**International Symposium on Chromatography and Mass Spectrometry in Nutrition Science and Food Safety**
To be held from 20 to 22 June in Montreux, Switzerland.
*Further information from the Italian Group for Mass Spectrometry in Biochemistry and Medicine, Via Eritrea 62, I 20157 Milan, Italy.*

**Medical Device & Diagnostic Industry (MD & DI) '83**
'Conference Expo' to be held from 20–22 June at the New York Hilton, USA.
*Further information from MD & DI '83, Expocon Management Associates, 163 Main Street, Westport, Connecticut 06880, USA.*

**European Committee for Clinical Laboratory Standards: Fourth Annual General Meeting and Fourth Seminar**
Covering 'Good Manufacturing Practice', 'Good Clinical Laboratory Practice', and 'The Value of Consultation between Clinicians, Surgeons and Laboratory Scientists'. To be held from 22–24 June at Trinity College, Dublin.
*Further information from Irene Batty, ECCLS Central Office, c/o Wellcome Research Laboratories, Langley Court, Beckenham, Kent BR3 3BS, UK.*

**ISA at Transducer/Tempcon 83**
The Instrument Society of America is organizing a two-day event at the Transducer/Tempcon exhibition and conference at the Wembley Conference Centre, London, from 28–30 June. The event will be subject to separate registration.
*Further information from Norma Thewlis, Trident International Exhibitions Ltd, 21 Plymouth Road, Tavistock, Devon PL19 8AU, UK.*

**IV International Meeting on Clinical Laboratory Organization and Management**
Organized by the Clinical Chemistry Data Communications Group and covering 'Optimized use of Clinical Laboratory Data'. To be held from 29 June to 1 July in Uppsala, Sweden.
*Further information from Uppsala 1983 CCDCG Conference, PO Box 2103, S-750 02 Uppsala, Sweden.*

### July 1983

**5th European Congress of Clinical Chemistry**
To be held from 3 to 8 July in Budapest, Hungary.
*Further information from MOTESZ Congress Bureau, PO Box 32, H 1361 Budapest, Hungary. Scientific correspondence to Dr P. Földvari, MAV Hospital, Central Laboratory, Rudas L u. III, H 1062 Budapest, Hungary.*

**Moisture Measurements in Solids and Gases: Available Techniques and Applications**
Course to be held at Sira Ltd, Chislehurst, UK from 5–6 July (fee = £160 + VAT).
*Further information from the Conference Unit, Sira Ltd, South Hill, Chislehurst, Kent BR7 5EH, UK.*

**Computers in Automation and Laboratory Management**
The fifth Summer School of Automatic Chemical Analysis. The course is residential and organized by Professor J. Betteridge, D. Porter and Dr P. Stockwell. To be held from 10 to 15 July at the University of Sussex, UK.
*Further information from Beverly Humphrey, 176a North View Road, London N8 7NB.*

**SAC 83: International Conference and Exhibition on Analytical Chemistry**
Organized by the Analytical Division of the Royal Society of Chemistry. To be held from 17–23 July at the University of Edinburgh.
*Further information from Miss P. E. Hutchinson, Analytical Division, Royal Society of Chemistry, Burlington House, London W1V 0BN.*

**Laboratory 83/SAC Exhibition**
To be held from 18–20 July at the University of Edinburgh, UK.
*Further information from Charles D. Cook, Erba Science (UK) Ltd, Headlands Trading Estate, Swindon, Wiltshire SN2 6JQ, UK.*

**American Association for Clinical Chemistry: Annual Meeting**
To be held from 24–29 July at Anaheim, USA.
*Further information from Sherago Convention Management, AACC Meeting Manager, 1515 Broadway, New York, New York 10036, USA.*

### August 1983

**Computing in Clinical Laboratories**
To be held from 24 to 26 August in Breda, The Netherlands.
*Further information from Mr R. C. J. Galle, Stichting Medische Laboratoria, Bergschot 69, 4817 PA Breda, The Netherlands.*

**American Chemical Society: Annual Meeting**
To be held from 28 August to 2 September in Washington, D.C.
*Contact ACS, 1155 16th Street, N.W., Washington, D.C. 20036.*

### September 1983

**Clinical Biochemistry Nearer the Patient**
To be held from 5–6 September at the University of Surrey, Guildford, UK.
*Further information from Professor Vincent Marks, Department of Clinical Biochemistry, University of Surrey, Guildford GU2 5XH, UK.*

**HRGC Forum 83**
To be held on 13 September at the University of Nottingham, UK.
*Further information from Charles D. Cook, Erba Science (UK) Ltd, Headlands Trading Estate, Swindon, Wiltshire SN2 6JQ, UK.*

**13th Philips X-ray Conference**
To be held from 19 to 23 September in Durham, UK.
*Further information from Maureen Courtney at Pye Unicam Ltd, York Street, Cambridge CB1 2PX, UK.*

**Columns in High Performance Liquid Chromatography**
To be held from 22–23 September, see below.

### October 1983

**Analyticon 83 and Laboratory 83**
To be held from 12 to 14 October at the Barbican Centre, London.
*Further information from Mr G. C. Young, SIMA (Scientific Instrument Manufacturers' Association of Great Britain), Leicester House, 8 Leicester Street, London WC2H 7BN.*

### November 1983

**Second African, Mediterranean and Near East Conference of Clinical Chemistry**
To be held from 20 to 24 November in Cairo.
*Further information from the Congress Secretariat, Second African, Mediterranean and Near East Congress of Clinical Chemistry, 6 Porter Street, Baker Street, London W1M IH2.*

### 1984 meetings

**XIIth International Congress of Clinical Chemistry**
To be held from 29 April to 4 May 1984 in Rio de Janeiro, Brazil.
*Further information from the Executive Secretary, XIIth International Congress, Rua Vicente Licinio 95, Cep 20270, Rio de Janeiro, Brazil.*

**2nd International Congress on Automation and New Technology in the Clinical Laboratory**
To be held from 15 to 18 October 1984 in Barcelona, Spain.
*For further information contact 2nd International Congress on Automation and New Technology in the Clinical Laboratory, IV Congreso Nacional de la Sociedad Española de Quimica Clinica, Apartado de Correos 543, Barcelona, Spain.*

**14th Annual Symposium on the Analytical Chemistry of Pollutants and the 3rd International Congress on Analytical Techniques in Environmental Chemistry**
To be held from 22–24 November 1984 at the Palacio de Congresos in Barcelona, Spain.
*For further information contact 14th Annual Symposium—3rd International Congress—Expoquimia, Avda. Reina M$^{a}$. Christina, Barcelona 4, Spain.*

### COLUMNS IN HIGH-PERFORMANCE LIQUID CHROMATOGRAPHY

A symposium on the topic 'Columns in High-Performance Liquid Chromatography' co-sponsored by the Wolfson L iquid Chromotography Unit, University of Edinburgh and Hewlett-Packard Ltd, Nine Mile Ride, Wokingham, Berkshire, will take place at the University of Cambridge from 22–23 September 1983 in the Lady Mitchell Hall.

The emphasis will be on:

Column thermodynamics, affecting
retention mechanisms and selectivity.
Column kinetics, affecting
separation efficiency.
Column geometry, affecting
speed of analysis, detection limit and aolvent consumption.

Presentations will be made in the form of discussion papers, as well as poster sessions.

The following scientists have already expressed an interest in actively participating in the symposium: E. Bayer (FR Germany), J. C. Berridge (UK), D. Dixon (FR Germany), H. Engelhardt (FR Germany), R. W. Frei (The Netherlands), N. R. Herbert (UK), H. Hulpke (FR Germany), K.-P. Hupe (FR Germany), R. Jonker (FR Germany), J. Kraak (The Netherlands), J. H. Knox (UK), W. Linder (Austria), M. Novotny (USA), P. J. Schoenmakers (The Netherlands). Further contributions are welcome—title and abstract should be submitted to Professor J. H. Knox, Department of Chemistry, University of Edinbrugh, West Mains Road, Edinburgh, UK, before 1 August 1983.

*Registration information (the symposium fee is £85·00) from Mrs Annet Pullen, Hewlett-Packard Ltd, Analytical Instrumentation, Nine Mile·Ride, Easthampstead, Wokingham, Berkshire RG11 3LL, UK. Tel.: 03446 3100, extension 3465.*

